# Association of LDL-C/HDL-C ratio with coronary heart disease: A meta-analysis

**DOI:** 10.1016/j.ihj.2024.01.014

**Published:** 2024-02-09

**Authors:** Siqi Hu, Hua Fan, Shenghui Zhang, Chen Chen, Yao You, Chunyi Wang, Jie Li, Lin Luo, Yongran Cheng, Mengyun Zhou, Xuezhi Zhao, Wen Wen, Tao Tan, Fangfang Xu, Xinyan Fu, Juan Chen, Xingwei Zhang, Mingwei Wang, Jiake Tang

**Affiliations:** a Department of Cardiology, Affiliated Hospital of Hangzhou Normal University, Hangzhou Institute of Cardiovascular Diseases, Zhejiang Key Laboratory for Research in Assessment of Cognitive Impairments, Hangzhou Normal University, Hangzhou, 310015, China; b Hangzhou Lin'an Fourth People's Hospital, Hangzhou, 311321, China; c School of Clinical Medicine, The First Affiliated Hospital of Henan University of Science and Technology, Henan University of Science and Technology, Luoyang, 471003, Henan, China; d Hangzhou Ruolin Hospital Management Co. Ltd, Hangzhou, 310007, China; e School of Public Health, Hangzhou Medical College, Hangzhou, 311300, China; f Department of Molecular & Cellular Physiology, Shinshu University School of Medicine, 3900803, Japan; g Department of Gynecology, Women's Hospital, School of Medicine, Zhejiang University, Hangzhou, 310006, Zhejiang, China; h Department of Cardiology, Huzhou Central Hospital, Affiliated Central Hospital of Huzhou University, 313000, Zhejiang, China; i Faculty of Applied Science, Macao Polytechnic University, Macao SAR, 999078, China; j Strategy Research and Knowledge Information Center, SAIC Motor Group, 200030, Shanghai, China

**Keywords:** Coronary heart disease, LDL-C to HDL-C ratio, Meta-analysis

## Abstract

**Background:**

Coronary heart disease (CHD) is a common heart disease and a leading cause of death in developed countries and some developing countries such as China. It is recognized as a multifactorial disease, with dyslipidemia being closely associated with the progression of coronary atherosclerosis. Numerous studies have confirmed the relationship between a single indicator of low-density lipoprotein cholesterol (LDL-C) or high-density lipoprotein cholesterol (HDL-C) and CHD. However, the association between LDL-C to HDL-C ratio (LHR) and CHD remains unclear. This study aimed to comprehensively explore the association between LHR and CHD.

**Methods:**

This meta-analysis was performed according to the Preferred Reporting Items for Systematic Reviews and Meta-analyses. PubMed, Embase, Web of Science, and China National Knowledge Infrastructure databases were comprehensively searched up to June 15, 2023, to find the studies that indicated the connection between LHR and CHD. A total of 12 published studies were selected. The random-effects model was used to pool the data and mean difference (MD), and the 95% confidence intervals (CI) were taken as the overall outcome. No language restrictions existed in the study selection. The Review Manager 5.4 and Stata 12 were used to analyze the data.

**Results:**

Twelve high-quality clinical studies involving 5544 participants, including 3009 patients with CHD, were enrolled in the meta-analysis. The findings revealed that the LHR was higher by 0.65 in patients with CHD than in those without CHD (MD, 0.65; 95% CI, 0.50–0.80).

**Conclusion:**

The LHR was found to be positively correlated with CHD, suggesting that it may serve as a potential indicator of CHD.

## 1 Introduction

Coronary heart disease (CHD) is a common heart disease and the leading cause of death in developed countries and some developing countries including China.^1^ It is recognized as a multifactorial disease, and dyslipidemia is closely associated with the progression of coronary atherosclerosis.^2^ Coronary atherosclerosis is the basis of coronary artery stenosis. Abnormal lipid metabolism, coagulation system, and inflammatory factor stimulation are some risk factors that damage endothelial cells, promote inflammatory reaction and lipid deposition, and thus accelerate plaque formation.^3^ Dyslipidemia accounts for about 50% of the population-attributable risk for CHD.^4^ Currently, we use anatomic criteria (e.g., the presence of at least one coronary stenosis) to establish the diagnosis of CHD by coronary angiography. The diagnosis of CHD was defined by the presence of a coronary artery stenosis of 50% or greater (by quantitative evaluation).^5^

At present, increases in plasma total cholesterol (TC), low-density lipoprotein cholesterol (LDL-C), non-high-density lipoprotein cholesterol (non-HDL-C), very low-density lipoprotein cholesterol (VLDL-C), triglyceride (TG), and/or lipoprotein (a) and decreases in HDL-C are commonly used as CHD predictors in clinical practice.^6^ For example, raised TC is a strong risk factor for CHD.^7^ Recent studies reported that when the conventional lipid parameters of TGs, namely HDL-C, LDL-C, and TC, remain apparently normal, other lipid parameters, such as lipid ratios, including TC/HDL-C, LDL-C/HDL-C, TG/HDL-C, and non-HDL-C/HDL-C, are the diagnostic alternatives predicting the risk of a cardiovascular event.^8^

A number of studies argue that the lipoprotein ratios, which have atherogenic components (such as TG, TC, and LDL-C) in the numerator and antiatherogenic components (such as HDL-C) in the denominator, could be better predictors for the occurrence^9^^,^^10^ and development of CHD than the individual lipid parameters. Specifically, the major anti-atherosclerotic effect of HDL-C is the reverse cholesterol transport. HDL-C scavenges cholesterol from the peripheral vasculature and transports it to the liver for excretion into the biliary system.^11^ Moreover, LDL-C is considered a classic atherogenic lipoprotein, and increased levels of LDL-C could transfer more cholesterol from the liver to the peripheral tissues, thereby increasing the risk of atherosclerosis.^12^ The LDL-C/HDL-C ratio (LHR) was found to be significantly associated with an increased risk of high carotid intima–media thickness in a Chinese cohort.^13^ However, a few studies have reported on the association between lipid ratios and CHD, and the evidence of the association between LHR and CHD is still limited. Therefore, we conducted a meta-analysis to comprehensively explore the association between LHR and CHD.

## 2 Materials and methods

### 2.1 Search strategy

The protocol for this meta-analysis adhered to the Preferred Reporting Items for Systematic Reviews and Meta-analyses (PRISMA) statements. We performed a comprehensive literature search using PubMed, Embase, Web of Science, and CNKI databases up to April 15, 2023. A search strategy involving both text and medical subject headings was employed, utilizing the keywords: “CHD” or “coronary heart disease” and “LDL-C/HDL-C” or “LDL-C to HDL-C ratio.” The search was confined to studies on human beings.

### 2.2 Inclusion criteria

The inclusion criteria of this meta-analysis were as follows: (1) prospective, cross-sectional, or retrospective design; (2) original studies evaluating the association between LHR and CHD; (3) the control group comprised healthy participants without other metabolic diseases; (4) diagnosis of CHD based on coronary angiography; (5) the age of patients ≥18 years; and (6) mean and standard deviation (SD)provided or could be calculated. The eligible studies were required to be published as peer review studies in full length without language restrictions. Duplicated reports, abstracts, letters/case reports, reviews, and editorials were excluded based on the title and abstract. Moreover, studies were also excluded if they did not furnish sufficient information to calculate the point estimate for the relevant outcome.

### 2.3 Study selection and data extraction

Two investigators (Siqi Hu and Yao You) independently screened all titles and abstracts from the retrieved studies, and reviewed the articles of the eligible studies. The relevant characteristics of included studies were extracted into an electronic database, including: (1) study details such as the first author, year of publication, location, and study design; (2) participants information, including number, age, and sex of the CHD and non-CHD groups; (3) diagnostic methods used for CHD; and (4) levels of LHR in the CHD group compared with the control group, among other details.

### 2.4 Quality assessment

The quality of the methodology of the included studies was assessed using the Newcastle–Ottawa Quality Assessment Scale (NOS). Each study received a quality score ranging from 1 to 9 stars, based on three key aspects: (1) selection of participants; (2) comparability of groups; and (3) exposure evaluation for case–control studies. Generally, studies that met at least five of the criteria were accepted as high-quality studies. Two reviewers (Siqi Hu and Yao You) independently assessed the quality of the original studies, and any disagreements were resolved with a third reviewer.

### 2.5 Statistical analysis

The Review Manager 5.4 and Stata 12 were used for the statistical analysis. Our meta-analysis used LHR as the primary outcome based on the random-effects model. LHR was presented as means ± SDs. Clinical heterogeneity among individual study estimates was assessed using the *χ*^2^ test and quantified by the *I*^2^ statistic, with values > 50% indicating a least moderate statistical heterogeneity. Meta-regression analyses were further carried out in case of significant heterogeneity. Sensitivity analysis was conducted by removing each study one at a time to assess its impact on the overall effect estimate. Funnel plots were generated to examine the presence of publication bias.

## 3 Results

### 3.1 Search results

**Fig. 1:**
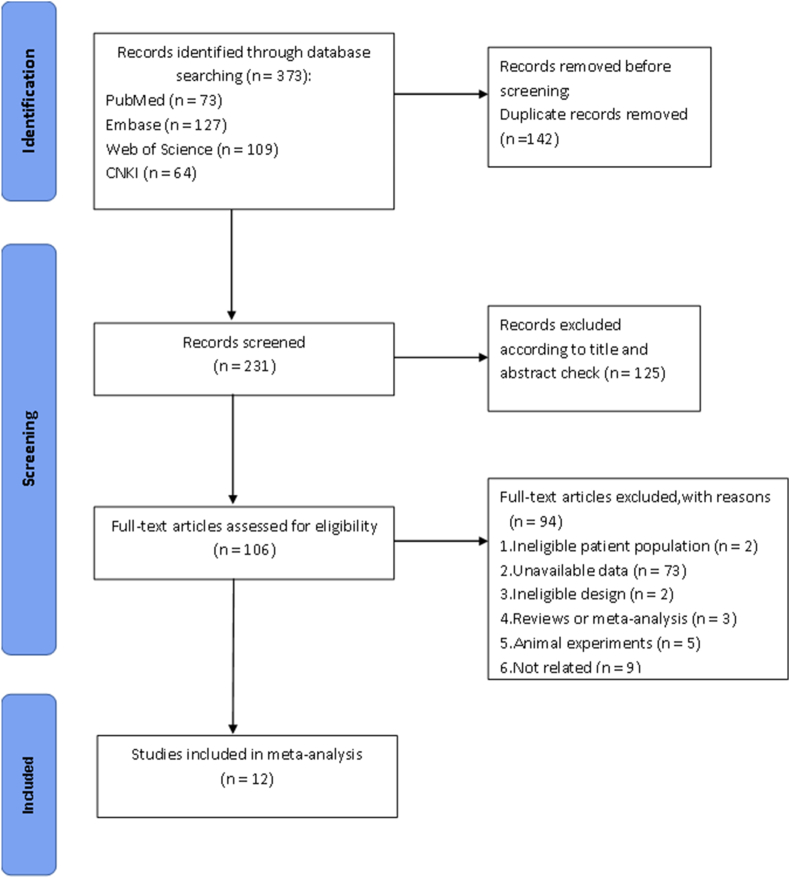
Flow diagram of the study selection process.

The PRISMA flowchart depicting the literature search process is illustrated (Fig. 1). The electronic and manual search resulted in the retrieval of 373 potentially relevant publications. After the initial screening, 125 studies were excluded based on the title and abstract review. Upon reviewing the full studies, an additional 94 studies were excluded because of (1) ineligible design or patient population; (2) unavailability of outcome data of interest; or (3) the study being an animal experiment, review, meta-analysis, or lacking relevance. Finally, 12 case–control studies were deemed eligible for inclusion in the meta-analysis.^6^^,^^14^, ^15^, ^16^, ^17^, ^18^, ^19^, ^20^, ^21^, ^22^, ^23^, ^24^

### 3.2 Baseline characteristics

The main characteristics of the included studies in this analysis are outlined in Table 1. Overall, our analysis included 12 studies enrolling 5544 participants (including 3009 patients with CHD). All studies were case–control studies, with sample sizes varying from 125 to 1351 participants. Except for one study in which the country could not be identified, all the other studies were conducted in China. Besides, all studies used coronary angiography for diagnosing CHD. Notably, most experiments collected 12-h fasting venous blood to detect LHR by enzymatic methods. In terms of quality assessment, the NOS scores indicated that the included studies were of high quality (Table 2).

**Table 1:** Characteristics of studies contained in the meta-analyses.

Author	Year	Location	Study Design	Diagnostic method	CHD				non-CHD			
					n	Male	Age	LDL-C/HDL-C	n	Male	Age	LHR
Iris Shai	2004	NM	Case-control	Coronary angiography	234	0	61.4 ± 6.6	3 ± 1.1	449	0	61.2 ± 6.6	2.4 ± 1
Li Zhu	2015	China	Case-control	Coronary angiography	738	527	63.23 ± 10.39	2.67 ± 1.7	157	83	59.29 ± 9.21	2.37 ± 0.87
Chao Ding	2015	China	Case-control	Coronary angiography	169	103	62.33 ± 9.07	2.85 ± 1.14	103	40	58.40 ± 9.27	2.2 ± 0.87
Meiyan Liu	2002	China	Case-control	Coronary angiography	106	73	63.5±lO.6	2.22 ± 0.72	92	48	56.1±l2.3	1.77 ± 0.63
Yandan Wu	2014	China	Case-control	Coronary angiography	175	116	28–80	2.74 ± 0.72	146	102	24–75	1.6 ± 0.45
Fengju Zhang	2008	China	Case-control	Coronary angiography	68	47	66.7 ± 6.5	2.31 ± 0.77	57	37	57.2 ± 9.7	1.76 ± 0.64
Ping Wang	2006	China	Case-control	Coronary angiography	43	NM	NM	6.2 ± 4.26	100	NM	20–79	2.98 ± 1.14
Jian Wang	2008	China	Case-control	Coronary angiography	199	127	56 ± 17	2.44 ± 0.41	189	110	53 ± 19	1.67 ± 0.3
Xiong Wang	2004	China	Case-control	Coronary angiography	132	65	38–79	2.24 ± 0.41	121	66	35–75	1.81 ± 0.3
Zhigang Lu	2002	China	Case-control	Coronary angiography	392	282	61.07 ± 8.75	3.67 ± 1.33	253	146	57.28 ± 9.99	2.76 ± 1.29
Yeming Ma	2009	China	Case-control	Coronary angiography	140	80	35–78	2.24 ± 0.42	130	88	18–65	1.81 ± 0.3
Ting Sun	2022	China	Case-control	Coronary angiography	613	372	66.51 ± 10.44	2.94 ± 1.06	738	324	66.51 ± 10.44	2.36 ± 0.78

**Table 2:** Quality assessment of included studies.

Authors	Year	Selection				Comparability		Outcome/exposure			Score
		1	2	3	4	5	6	7	8	9	
eChao Ding	2015	*	*	*	*	*	*	*	*		8
Fengju Zhang	2008	*	*	*	*	*		*	*		7
Iris Shai	2004	*	*	*	*	*		*	*	*	8
Jian Wang	2008	*	*		*	*	*	*	*		7
Li Zhu	2015	*	*	*	*	*	*	*	*		8
Meiyan Liu	2002	*	*	*	*	*	*	*	*		8
Ping Wang	2006	*	*	*	*	*		*	*		7
Xiong Wang	2004	*	*		*			*	*		5
Yandan Wu	2014	*	*	*	*	*	*	*	*		8
Yeming Ma	2009	*	*		*			*	*		5
Zhigang Lu	2002	*	*	*	*			*	*		6
Ting Sun	2022	*	*	*	*	*	*	*	*		8

### 3.3 Association between LHR and CHD

**Fig. 2:**
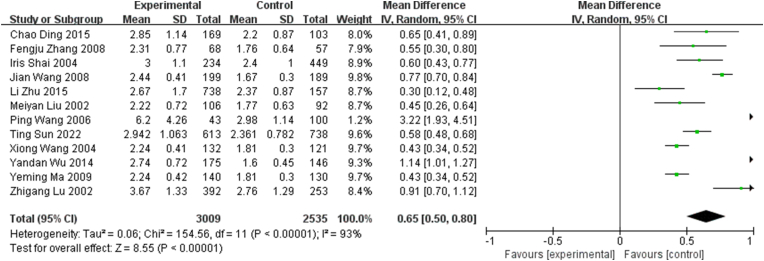
Meta-analysis of studies reporting LHR in coronary heart disease (CHD) vs. non-CHD subjects using a random-effects model and a mean difference (MD) with 95% confidence intervals (CIs).

All the 12 studies included continuous LHR data to quantitatively evaluate the association between LHR and CHD (Fig. 2). The LHR was higher by 0.65 on an average in patients with CHD compared with those without CHD [mean difference (MD), 0.65; 95% confidence interval (CI), 0.50–0.80)] (*I*^2^ = 93%, *P* for heterogeneity <0.01). Regarding statistical heterogeneity, we found evidence of heterogeneity across the included studies (*I*^2^ = 93%, *P* for heterogeneity <0.01).

### 3.4 Association between TG/HDL-C and CHD

**Fig. 3:**
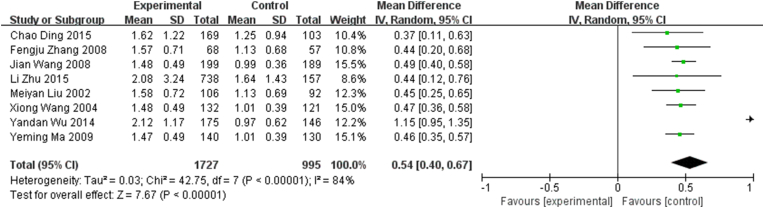
Meta-analysis of studies reporting TG/HDL-C in coronary heart disease (CHD) vs. non-CHD subjects using a random-effects model and a mean difference (MD) with 95% confidence intervals (CIs).

Of the 12 studies included, 8 provided continuous TG/HDL-C data to quantitatively evaluate the association between TG/HDL-C and CHD (Fig. 3). The TG/HDL-C ratio was higher by 0.54 on an average in patients with CHD compared with those without CHD (MD, 0.54; 95% CI, 0.40–0.67) (*I*^2^ = 84%, *P* for heterogeneity <0.01). Regarding statistical heterogeneity, we found evidence of heterogeneity across the included studies (*I*^2^ = 84%, *P* for heterogeneity <0.01).

### 3.5 Meta-regression analysis

Meta-regression showed that the year of publication and the proportion of males were not sources of heterogeneity.

### 3.6 Sensitivity analysis

**Fig. 4:**
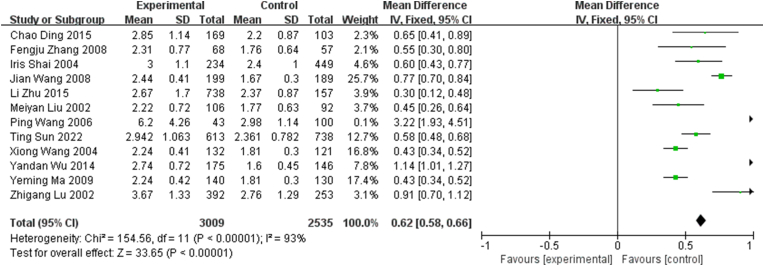
Meta-analysis of studies reporting LDL-C-TO-HDL-C in coronary heart disease (CHD) vs. non-CHD subjects using a fixed-effects model and a mean difference (MD) with 95% confidence intervals (CIs).

Furthermore, exclusion sensitivity analysis did not affect the overall estimate. The results were invariant when the fixed-effects model was selected, with MD = 0.62 (95% CI, 0.58–0.66) (Fig. 4).

### 3.7 Publication bias

**Fig. 5:**
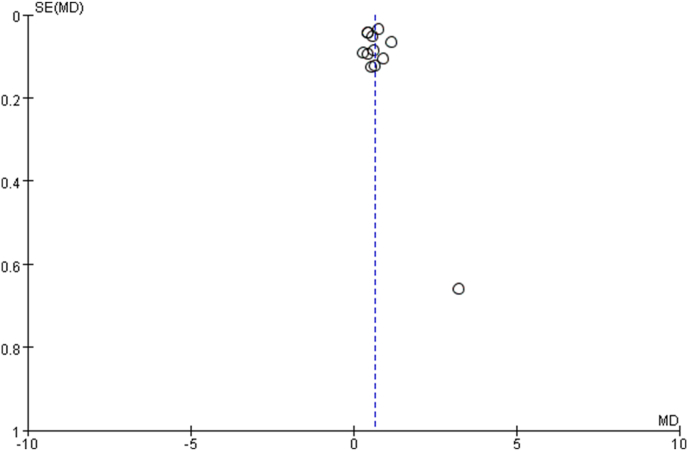
Funnel plot of meta-analysis.

The funnel figure is shown in Fig. 5. The overall plot was symmetric, indicating a low possibility of publication bias. In addition, the Begg's test (*p* = 0.304) and Egger's test (*p* = 0.559) were also conducted, and both tests confirmed no publication bias.

## 4 Discussion

Twelve high-quality clinical studies, 5544 participants, and 3009 patients with CHD were included in this study. We concluded that the LHR was higher by 0.65 in patients with CHD compared with those without CHD. Incidentally, we found that the TG/HDL-C level was higher by 0.54 in patients with CHD compared with those without CHD. The risk of CHD does not suddenly increase with the existence of stenosis but reflects the burden of disease in a wide range, with the degree of atherosclerotic disease rising from very low to very high.^25^ The pathological basis of CHD was mainly atherosclerosis and abnormal lipid metabolism. A lipid triad involving atherogenic dyslipidemia characterized by moderate/high LDL-C, low HDL-C, and elevated TG occurs in numerous clinical settings associated with a high cardiovascular risk.^26^ LDL-C/HDL-C is clinically regarded as the atherosclerosis index. Both Gerben's and Procam's heart studies indicated that the basic level of LHR was positively correlated with the risk of CHD.^27^ While previous studies distinguished the degree of coronary artery disease in patients, we only classified patients with or without CHD. Yang et al showed that the severity of coronary artery lesions was related to abnormal lipid metabolism, suggesting that LHR and TC/HDL-C were better indicators.^28^ The association between LHR and CHD was still unclear, so we chose this ratio index for full retrieval and discussion.

The nomenclature for defining CHD has been controversial, with terms such as coronary artery disease, coronary heart disease, and ischemic heart disease often used interchangeably. For example, the American Heart Association guidelines on treating serum cholesterol to reduce adult cardiovascular risk involve clinical atherosclerotic cardiovascular diseases, including CHD.^29^ Many studies showed that lipid metabolism disorder played a crucial role in the occurrence of CHD. This study aimed to explore the association between lipid metabolism and CHD. Previous studies showed that high TG alone was not an independent risk factor for CHD; rather, it becomes significant when accompanied by high TC, high LDL-C, and low HDL-C.^30^ On a continuous scale, a 1 − SD (32 mg/dL) difference in LDL-C was associated with a 40% increase in the risk of CHD.^14^ Moreover, people with higher HDL-C levels exhibit a significantly lower risk of CHD. Iris et al found that a 1 − SD difference in HDL-C (17 mg/dL) was associated with a 67% increase in CHD risk.^14^ Therefore, we aimed to investigate whether LHR increases in CHD and whether this index can be used to predict CHD. Although many studies have evaluated the risk of CHD using a single index, the evaluation is not comprehensive. Clinical studies showed that despite lowering LDL-C, serious adverse cardiovascular events may persist, and residual cardiovascular risks may be related to lipid abnormalities, especially those causing atherosclerosis.^31^ The relationship between individual indexes of blood lipids is not isolated, and predicting the risk of CHD with only one index is not enough. The lipoprotein ratios have atherogenic components (e.g., LDL-C) in the numerator and antiatherogenic components (e.g., HDL-C) in the denominator. The results of LHR are consistent with those of single indexes, all obtained by extracting and separating serum from fasting venous blood. LHR is more sensitive and comprehensive than a single blood lipid index. The LHR level objectively reflects heterogeneous changes in CHD.

The TG/HDL-C ratio was significantly higher in patients with CHD than in the normal group, and the ratio was predictive of the severity of CHD in the study from Ruijin Hospital in China.^32^ Similarly, it was also a ratio index, and this research also proved that LHR could increase in people with CHD. LHR was closely correlated with the distribution of HDL-C subclasses. As the ratio increased, small-sized pre-β1-HDL, HDL3b, and HDL3a increased, but large-sized HDL2a and HDL2b decreased. This hampered the reverse cholesterol transport from peripheral tissues to the liver and steroidogenic organs and induced atherosclerosis progression.^33^ Therefore, LHR could be used as a predictive index, which could detect high-risk patients and intervene reasonably as soon as possible, and had certain clinical practical significance for preventing the occurrence and development of atherosclerosis.

In addition, we conducted a sensitivity analysis by omitting one study at a time, and the results remained stable. No significant changes were observed after excluding any of the included studies. Similarly, even when using the fixed-effects model for analysis, the results did not change significantly. Further, no obvious publication bias was found in this study, and the value of most studies was within a reasonable range according to the funnel plot results. The relatively small sample size inevitably introduced selection bias, and the single-center nature of some included studies may lead to a potential overestimation of the effect.

This study integrated the research on the association between LHR and CHD during the last 20 years. The findings suggested that LHR could provide new clues for predicting CHD. However, this study still has some limitations. First, three English databases were searched, and most of the collected data were from China due to a limited number of related studies. Therefore, it is uncertain whether these results can be applied to other countries. Future studies should include data from other countries to draw comprehensive conclusions. Second, the research results had certain heterogeneity. We only included case–control studies, and the included studies did not classify specific types of CHD. We collected data from a single study on people with special physiological characteristics (postmenopausal women), making it difficult to perform meaningful subgroup analysis. We intend to conduct more comprehensive, centralized, and forward-looking research to confirm these findings. Finally, we performed a meta-regression analysis to determine the source of heterogeneity, and the results showed that the publication year and sex were not the sources of heterogeneity. Most of the included studies did not collect data on unknown or unmeasured risk factors (such as body mass index, smoking, lifestyle, eating habits), and information on whether patients with CHD took lipid-lowering drugs. These factors could significantly impact LHR. In addition, heterogeneity may also be attributed to different center settings, time constraints, and other reasons.

In conclusion, the results of this study proved that LHR could increase the risk of CHD. From a clinical point of view, a high LHR may be a possible risk factor for CHD, and this hypothesis was reasonable. Patients with a high LHR may need to pay attention to and promptly control their blood lipid levels to prevent CHD because of the high prevalence and mortality of CHD. From a public health perspective, a large-scale prospective study should be conducted to further verify this association and clarify the connection between LHR and the pathogenesis of CHD. Meanwhile, further interventions in LHR testing are needed to determine whether dietary or drug interventions can reduce the occurrence and progression of CHD.

## Funding

This study was supported by 10.13039/501100003786Hangzhou Science and Technology Bureau fund (No. 20191203B96; No. 20191203B105); Youth Fund of Zhejiang 10.13039/501100000691Academy of Medical Sciences (No. 2019Y009); Medical and Technology Project of Zhejiang Province (No. 2021KY890; No. 2024KY1348; No. 2024KY200); Zhejiang Traditional Chinese Medicine Scientific Research Fund Project (No. 2022ZB280; No. 2024ZL723); Zhejiang Kangenbei Hospital Management Soft Science Research Project (No.2022ZHA-KEB316); Hospital Fund of Affiliated Hospital of 10.13039/501100007820Hangzhou Normal University (No.2021YN2021112); Hangzhou Agricultural and Social Development Scientific Research Guidance Project (No.20220919Y021). The funders have no role in the data collection, data analysis, preparation of manuscript and decision to submission. The work was supported by the Key medical disciplines of Hangzhou.

## Data sharing statement

All the data and materials mentioned in the manuscript are available.

## Declaration of competing interest

The authors declare that they have no known competing financial interests or personal relationships that could have appeared to influence the work reported in this paper.
